# Diabetes and Mortality From Respiratory Diseases: The Japan Collaborative Cohort Study

**DOI:** 10.2188/jea.JE20190091

**Published:** 2020-10-05

**Authors:** Mengying Wang, Isao Muraki, Keyang Liu, Kokoro Shirai, Akiko Tamakoshi, Yonghua Hu, Hiroyasu Iso

**Affiliations:** 1 School of Public Health, Peking University, Beijing, China; 2 Public Health, Department of Social Medicine, Osaka University Graduate School of Medicine, Osaka, Japan; 3 Department of Public Health, Faculty of Medicine Hokkaido University, Sapporo, Japan

**Keywords:** diabetes, mortality, respiratory diseases, cohort studies

## Abstract

**Background:**

Little evidence is available about the association between diabetes and respiratory disease mortality among Japanese populations. We aimed to explore the association between diabetes and the risk of respiratory diseases mortality through a nationwide prospective study in Japan.

**Methods:**

We followed 95,056 participants (39,925 men and 55,131 women) for a median 17.1 years. The information about diabetes status, sociodemographic characteristics, and lifestyles was collected at baseline. Cox proportional hazards regression models were used to estimate hazard ratios (HRs) of mortality from respiratory diseases associated with baseline diabetes status.

**Results:**

We identified 2,838 deaths from total respiratory diseases (1,759 respiratory infection, 432 chronic obstructive pulmonary disease, and 647 other respiratory diseases). The association between diabetes and total respiratory disease mortality was statistically significant among women (HR 1.81; 95% CI, 1.39–2.37) but of borderline statistical significance in men (*P* for interaction <0.01). Besides, there were significant associations between diabetes and mortality from respiratory infection among both men and women (HR 1.39; 95% CI, 1.10–1.76 and HR 2.30; 95% CI, 1.71–3.11, respectively; *P* for interaction <0.001). However, we failed to detect any statistically significant association between diabetes and COPD mortality. Moreover, the subgroup analysis revealed that the association between diabetes and total respiratory disease mortality was stronger in never smokers when compared with ever smokers (*P* for interaction = 0.02).

**Conclusions:**

Significant association was observed between diabetes and the risk of total respiratory disease mortality, in particular from respiratory infection. Prevention and control of respiratory diseases, especially respiratory infection, should be paid more attention among people with diabetes in clinical and public health practice.

## INTRODUCTION

As an important risk factor for cardiovascular diseases, diabetes has significant impact on global health, and contributed to 1.5 million deaths in 2012 worldwide.^1^ In addition, diabetes is responsible for the morbidity and mortality of a number of diseases including stroke, ischemic heart diseases, and cancer.^2^^–^^4^ In Japan, the prevalence of diabetes has increased rapidly over the last decades.^5^ It is estimated that approximately 7.2 million Japanese people had diabetes in 2013.^6^

Although several studies have showed that clinical outcomes in patients with diabetes have improved over time, diabetes-related complications have risen due to the increased prevalence of the disease.^7^^,^^8^ Thus, the influence of diabetes on the disease-related outcomes still needs further study. Previous studies have demonstrated associations between diabetes and risk of mortality from respiratory diseases, such as chronic obstructive pulmonary disease (COPD), pneumonia, and acute respiratory distress syndrome (ARDS),^9^^–^^11^ yet the results were inconclusive among different studies. Bragg et al^9^ found that diabetes was significantly associated with increased mortality from chronic respiratory diseases, mainly COPD, through a nationwide prospective study of 512,869 adults in China (rate ratio 1.29; 95% CI, 1.10–1.51). An Australian prospective study which enrolled 1,108,982 individuals with diabetes indicated that standardized pneumonia mortality was 1.22-fold (95% confidence interval [CI], 1.17–1.27) higher in those with type 2 diabetes compared with general populations.^10^ Moreover, Soubani et al^11^ conducted a retrospective cohort study of ARDS patients, which showed that diabetes may not have effect on the mortality of ARDS patients. However, to our knowledge, little evidence was found about whether presence of diabetes was associated with higher mortality from respiratory diseases among Japanese populations. Since respiratory disease, especially pneumonia, is one of the leading causes of deaths among Japanese elderly people,^12^ it is important to evaluate the association between diabetes and the risk of mortality due to respiratory disease for better prevention and control of the diseases in Japan.

The aim of the study was to explore the association between self-reported diabetes and the mortality due to respiratory diseases through a nationwide prospective study in Japan.

## METHODS

### Study design and participants

The Japan Collaborative Cohort Study is a nationwide population-based cohort study, with the study design and methods described in detail previously.^13^ Briefly, a number of 110,585 participants (46,395 men and 64,190 women) aged 40–79 years were recruited in the baseline survey from 45 communities in Japan during 1988 through 1990. In most of the 45 areas, the follow-up was completed by the end of 2009, while the follow-up was completed at the end of 2008 in two areas. Besides, the follow-up was stopped at the end of 2003 and 1999 in four areas. When individuals declared their participation in the study by choosing “agree to participate in this study” for the first question in baseline questionnaire, we considered that informed consent was valid. In several communities, informed consent was obtained from leaders of local government level.^14^ The study was approved by the Ethics Committees of the Nagoya University School of Medicine and Osaka University.

In the current study, the participants who failed to answer the question about their diabetic status at baseline were excluded. The remaining 95,056 participants (39,925 men and 55,131 women) were included in the analysis.

### Data collection

All participants were required to complete a baseline self-administered questionnaire to collect information about sociodemographic characteristics, lifestyles, and medical history of prevalent diseases at baseline.

The question about the history of diabetes in the baseline questionnaire was “Have you had physician-diagnosed diabetes? (No/Yes, being treated/Yes, received treatment previously/Yes, without treatment)”. Previously diagnosed diabetes was defined by choosing any of the last three answers.

Information on tobacco smoking was obtained through asking the participants to describe their smoking status: never, former, or current, where former smokers and current smokers were defined as ever smokers. In addition, drinking status was asked to classify subjects into never-, ex-, and current drinkers. Moreover, information of height and weight was obtained from the self-reported questionnaire. Body mass index (BMI) was calculated as weight in kilograms divided by the square of height in meters and classified into five groups of BMI <18.5 kg/m^2^, BMI 18.5–21.9 kg/m^2^, BMI 22.0–24.9 kg/m^2^, BMI 25.0–26.9, and BMI ≥27.0 kg/m^2^.

### Ascertainment of outcomes

Death date and cause of death were obtained by reviewing the death certificate and coded according to the International Classification of Disease, 10th revised edition (ICD-10).^14^ The outcomes of this study were the primary cause of deaths due to respiratory diseases, defined according to ICD-10 code as J00–J99, where COPD and respiratory infection were identified as code of J40–J47 and J00–J22, respectively.

### Statistical analysis

Student’s *t*-test was used to test the differences between different baseline diabetic history groups for continuous variables, while the Chi-square test was used to compare percentages of categorical variables between different baseline diabetic history groups.

Person-years for each participant were calculated as the duration from the response date of baseline survey through death date or date of lost to follow-up, whichever came first. The Cox proportional hazards regression model was adopted to calculate hazard ratios (HRs) of mortality from respiratory diseases. In the first model, all HRs were adjusted for age at baseline. We also adopted multivariable model to adjust for the potential confounding factors, namely, education level (attending school after 18 years or not), BMI (<18.5, 18.5–21.9, 22.0–24.9, 25–26.9, or >27 kg/m^2^), sports activity time (never, 1–2 hours/week, 3–4 hours/week, or ≥5 hours/week), average daily walking time (never, 0.5 hour/day, 0.5–1 hour/day, or ≥1 hour/day), smoking status (never smokers, ex-smokers, current smokers), alcohol use (never drinkers, ex-drinkers, current drinkers), and family history of diabetes (yes/no). In addition, tests for interaction were carried out with Cox proportional hazards regression analyses by setting variable cross-product terms of diabetes with sex, age (<65 and ≥65 years), smoking (never smoking and ever smoking), and BMI (<25 and ≥25 kg/m^2^) in the model.

All analyses were performed using SAS software (version 9.4; SAS Institute Inc., Cary, NC, USA). All *P*-values for the tests were two-sided and *P*-values <0.05 were considered statistically significant.

## RESULTS

Of the 95,056 participants (aged 57.1 [standard deviation, 10.1] years), the prevalence of self-reported diabetes was 4.5% (3.6% of women and 5.8% of men). Diabetic patients were older than non-diabetic participants at baseline. The percentage of obesity was higher among diabetic patients when compared to non-diabetic participants among both men and women. In addition, diabetic patients had less alcohol consumption and walking time but more sports activity than non-diabetic participants (Table 1).

**Table 1.** Sex-specific characteristics of the study variables according to baseline history of diabetes

	Men			Women		

	Baseline history of diabetes			Baseline history of diabetes		
	Yes	No	*P*-value	Yes	No	*P*-value
Number of participants	2,294	37,631		689	54,442	
Age, years	61.0 (9.0)	56.7 (10.2)	<0.001	63.2 (9.0)	57.0 (10.1)	<0.001
Education, %			<0.001			0.82
≤18 years	80.3	87.0		92.5	92.3	
>18 years	19.7	13.0		7.5	7.7	
Tobacco smoke, %			0.78			<0.001
Nonsmoker	17.3	21.3		90.3	93.4	
Ex-smoker	32.8	25.3		2.8	1.5	
Current smoker	49.9	53.4		6.9	5.1	
Alcohol use, %			0.02			<0.001
Nondrinker	17.4	18.8		78.6	74.3	
Ex-drinker	12.3	5.3		3.9	1.4	
Current drinker	70.3	75.9		17.5	24.3	
Sports activity, %			<0.001			<0.001
≥5 hours/week	9.2	7.1		6.8	4.5	
3–4 hours/week	7.9	7.1		6.2	5.3	
1–2 hours/week	19.2	16.9		15.0	13.7	
Never	63.7	68.9		72.0	76.5	
Walking time, %			<0.001			<0.001
≥1 hour/day	41.4	49.8		40.5	51.5	
0.5–1 hour/day	20.4	19.5		23.0	20.3	
0.5 hour/day	24.1	18.3		24.1	17.1	
Never	14.1	12.4		12.4	11.1	
BMI, kg/m^2^, %			0.04			<0.001
BMI <18.5	6.1	5.2		6.0	6.1	
18.5 ≤ BMI < 22.0	34.5	36.9		28.8	33.8	
22.0 ≤ BMI < 25.0	38.4	39.4		36.4	37.5	
25.0 ≤ BMI < 27.0	13.4	12.1		14.6	13.2	
BMI ≥27.0	7.6	6.4		14.2	9.4	

BMI, body mass index.

During the median follow-up of 17.1 years, we identified 2,838 deaths from total respiratory diseases (1,759 respiratory infection, 432 COPD, and 647 other respiratory diseases). In both the age-adjusted model and multivariable-adjusted model adjusting for educational level, BMI, sports activity, walking, smoking, alcohol consumption, and family history of diabetes, we failed to detect statistically significant association between diabetes and the risk of mortality from total respiratory diseases among men (*P* > 0.05). However, in the age-adjusted model, diabetic women had a higher risk of mortality from total respiratory diseases when compared to those without diabetes (HR 1.70; 95% CI, 1.31–2.21) (Table 2). After adjustment for other potential risk factors, the association remained statistically significant (HR 1.81; 95% CI, 1.39–2.37) (Table 2). Thus, sex may significantly modify the association between diabetes and risk of mortality from total respiratory diseases (*P* for interaction <0.01). Moreover, there was significant association between diabetes and the risk of mortality due to respiratory infection among both men and women in the age-adjusted model (HR 1.36; 95% CI, 1.08–1.72 in men and HR 2.11; 95% CI, 1.58–2.83 in women) as well as multivariable-adjusted model (HR 1.39; 95% CI, 1.10–1.76 in men and HR 2.30; 95% CI, 1.71–3.11 in women) (Table 2). However, for COPD and other respiratory diseases, diabetes was not associated with the risk of mortality among either men or women (Table 2).

**Table 2.** Sex-specific and age-adjusted and multivariable hazard ratios (HRs) of mortality from respiratory diseases according to baseline history of diabetes

	Men			Women			

	Baseline history of diabetes			Baseline history of diabetes			*P*_interaction_
	Yes	No	*P* trend	Yes	No	*P* trend	
Person-years	25,829	553,686		17,774	855,010		
Total respiratory diseases							
Deaths	114	1,685		60	979		
HR^a^ (95% CI)	1.16 (0.96–1.41)	1.00	0.12	1.70 (1.31–2.21)	1.00	<0.001	<0.001
HR^b^ (95% CI)	1.20 (0.99–1.45)	1.00	0.07	1.81 (1.39–2.37)	1.00	<0.001	<0.01
Respiratory infection							
Deaths	78	976		49	656		
HR^a^ (95% CI)	1.36 (1.08–1.72)	1.00	0.01	2.11 (1.58–2.83)	1.00	<0.001	<0.001
HR^b^ (95% CI)	1.39 (1.10–1.76)	1.00	0.01	2.30 (1.71–3.11)	1.00	<0.001	<0.001
COPD							
Deaths	16	320		1	95		
HR^a^ (95% CI)	0.85 (0.52–1.41)	1.00	0.53	—	—	—	
HR^b^ (95% CI)	0.89 (0.53–1.47)	1.00	0.64	—	—	—	
Other respiratory diseases							
Deaths	20	389		10	228		
HR^a^ (95% CI)	0.91 (0.58–1.43)	1.00	0.68	1.21 (0.64–2.29)	1.00	0.55	0.05
HR^b^ (95% CI)	0.96 (0.61–1.52)	1.00	0.87	1.22 (0.64–2.33)	1.00	0.54	0.81

CI, confidence interval; COPD, chronic obstructive pulmonary disease; HR, hazard ratio.

^a^Adjusted for age.

^b^Adjusted for age, educational level, body mass index, smoking, alcohol consumption, sports activity, walking time, family history of diabetes.

In the subgroup analysis based on baseline age, we found that, for both people aged 40–64 years and 65–79 years, diabetic patients had an increased risk of mortality from total respiratory diseases (HR 1.97; 95% CI, 1.46–2.67 in those aged 40–64 years and HR 1.35; 95% CI, 1.12–1.62 in those aged 65–79 years; *P* for interaction <0.01) and mortality due to respiratory infection (HR 2.76; 95% CI, 1.93–3.95 in those aged 40–64 years and HR 1.58; 95% CI, 1.28–1.97 in those aged 65–79 years; *P* for interaction <0.001) in the multivariable-adjusted model (Table 3).

**Table 3.** Multivariable hazard ratios of mortality from respiratory diseases according to baseline history of diabetes, stratified by age groups

	Age <65 years			Age ≥65 years			

	Baseline history of diabetes			Baseline history of diabetes			*P*_interaction_
	Yes	No	*P* trend	Yes	No	*P* trend	
Person-years	31,231	1,075,394		16,082	2,702,145		
Total respiratory diseases							
Deaths	46	785		128	1,879		
HR^a^ (95% CI)	1.97 (1.46–2.67)	1.00	<0.001	1.35 (1.12–1.62)	1.00	0.01	<0.01
Respiratory infection							
Deaths	4	135		93	1,196		
HR^a^ (95% CI)	2.76 (1.93–3.95)	1.00	<0.001	1.58 (1.28–1.97)	1.00	<0.001	<0.001
COPD							
Deaths	34	436		13	280		
HR^a^ (95% CI)	0.92 (0.34–2.52)	1.00	0.87	0.82 (0.47–1.45)	1.00	0.50	<0.01
Other respiratory diseases							
Deaths	8	214		22	403		
HR^a^ (95% CI)	1.20 (0.59–2.45)	1.00	0.62	1.09 (0.71–1.69)	1.00	0.70	<0.001

CI, confidence interval; COPD, chronic obstructive pulmonary disease; HR, hazard ratio.

^a^Adjusted for sex, educational level, body mass index, smoking, alcohol consumption, sports activity, walking time, family history of diabetes.

We also conducted a subgroup analysis according to baseline smoking status. For never smokers, there was statistically a significant association between diabetes and the risk of mortality from total respiratory diseases (HR 1.71; 95% CI, 1.32–2.21) while the association was non-significant among ever smokers (*P* for interaction = 0.02). In addition, diabetes was associated with higher risk of mortality from respiratory infection among never smokers (HR 1.98; 95% CI, 1.47–2.66) than ever smokers (HR 1.40; 95% CI, 1.01–1.81; *P* for interaction <0.01) in the multivariable-adjusted model (Table 4).

**Table 4.** Age-adjusted and multivariable hazard ratios (HRs) of mortality from respiratory diseases according to baseline history of diabetes, stratified by smoking status

	Never smokers			Ever smokers^a^			

	Baseline history of diabetes			Baseline history of diabetes			*P*_interaction_
	Yes	No	*P* trend	Yes	No	*P* trend	
Person-years	21,412	786,742		21,685	459,772		
Total respiratory diseases							
Deaths	65	1,017		93	1,413		
HR^b^ (95% CI)	1.75 (1.36–2.25)	1.00	<0.001	1.12 (0.90–1.38)	1.00	0.31	<0.001
HR^c^ (95% CI)	1.71 (1.32–2.21)	1.00	<0.001	1.16 (0.93–1.43)	1.00	0.18	0.02
Respiratory infection							
Deaths	50	700		65	797		
HR^b^ (95% CI)	1.98 (1.48–2.64)	1.00	<0.001	1.37 (1.06–1.77)	1.00	0.01	<0.001
HR^c^ (95% CI)	1.98 (1.47–2.66)	1.00	<0.001	1.40 (1.01–1.81)	1.00	0.01	<0.01
COPD							
Deaths	4	92		12	289		
HR^b^ (95% CI)	1.11 (0.41–3.02)	1.00	0.84	0.69 (0.39–1.23)	1.00	0.21	0.12
HR^c^ (95% CI)	1.01 (0.37–2.82)	1.00	0.98	0.75 (0.42–1.34)	1.00	0.33	0.70
Other respiratory diseases							
Deaths	11	225		16	327		
HR^b^ (95% CI)	1.35 (0.73–2.47)	1.00	0.34	0.86 (0.52–1.42)	1.00	0.55	0.10
HR^c^ (95% CI)	1.26 (0.68–2.34)	1.00	0.46	0.90 (0.54–1.50)	1.00	0.68	0.94

CI, confidence interval; COPD, chronic obstructive pulmonary disease; HR, hazard ratio.

^a^Former smokers and current smokers.

^b^Adjusted for age.

^c^Adjusted for age, sex, educational level, body mass index, alcohol consumption, sports activity, walking time, family history of diabetes.

In order to explore whether the association was modified by BMI levels, we conducted another subgroup analysis according to baseline BMI (<25 kg/m^2^ and ≥25 kg/m^2^), which showed that diabetic patients were more likely to die of total respiratory diseases (HR 1.63; 95% CI, 1.08–2.45) in the multivariable-adjusted model among participants with BMI ≥25 kg/m^2^ when compared with participants with BMI <25 kg/m^2^ (HR 1.19; 95% CI, 0.99–1.43). Further, a significant association was observed between diabetes and risk of mortality due to respiratory infection among participants with baseline BMI <25 kg/m^2^ (HR 1.48; 95% CI, 1.19–1.84) and those with BMI ≥25 kg/m^2^ (HR 1.82; 95% CI, 1.12–2.98; *P* for interaction = 0.54) (Table 5).

**Table 5.** Age-adjusted and multivariable hazard ratios (HRs) of mortality from respiratory diseases according to baseline history of diabetes, stratified by body mass index

	Body mass index <25 kg/m^2^			Body mass index ≥25 kg/m^2^			

	Baseline history of diabetes			Baseline history of diabetes			*P*_interaction_
	Yes	No	*P* trend	Yes	No	*P* trend	
Person-years	33,391	1,006,460		11,541	273,969		
Total respiratory diseases							
Deaths	125	2,063		27	323		
HR^a^ (95% CI)	1.39 (1.16–1.66)	1.00	<0.001	1.66 (1.12–2.46)	1.00	0.01	0.78
HR^b^ (95% CI)	1.19 (0.99–1.43)	1.00	0.06	1.63 (1.08–2.45)	1.00	0.02	0.99
Respiratory infection							
Deaths	92	1,255		19	203		
HR^a^ (95% CI)	1.68 (1.36–2.08)	1.00	<0.001	1.90 (1.19–3.05)	1.00	0.01	0.39
HR^b^ (95% CI)	1.48 (1.19–1.84)	1.00	<0.001	1.82 (1.12–2.98)	1.00	0.02	0.54
COPD							
Deaths	11	344		4	31		
HR^a^ (95% CI)	0.72 (0.40–1.32)	0.29	0.29	2.15 (0.76–6.12)	1.00	0.15	0.98
HR^b^ (95% CI)	0.56 (0.31–1.03)	1.00	0.06	2.13 (0.72–6.31)	1.00	0.17	0.88
Other respiratory diseases							
Deaths	22	464		4	89		
HR^a^ (95% CI)	1.09 (0.71–1.68)	1.00	0.68	0.91 (0.33–2.49)	1.00	0.86	0.44
HR^b^ (95% CI)	0.95 (0.62–1.47)	1.00	0.82	0.93 (0.33–2.57)	1.00	0.88	0.41

CI, confidence interval; COPD, chronic obstructive pulmonary disease; HR, hazard ratio.

^a^Adjusted for age.

^b^Adjusted for age, sex, educational level, smoking, alcohol consumption, sports activity, walking time, family history of diabetes.

## DISCUSSION

In the present prospective study of 95,056 Japanese populations, diabetes was significantly associated with higher mortality from total respiratory diseases among women rather than men. However, we found a significant association between diabetes and increased mortality due to respiratory infection among both men and women. The study indicated that diabetes was associated with risk of mortality from respiratory disease, mainly due to respiratory infection.

A number of studies have explored the association between diabetes and the risk of mortality from respiratory disease.^9^^,^^15^^,^^16^ A cohort study of 0.5 million Chinese people found that diabetes was associated with increased risk of mortality from total respiratory diseases (RR 1.29; 95% CI, 1.10–1.51) and pneumonia (RR 2.47; 95% CI, 1.80–3.38).^9^ In addition, Wright et al^15^ conducted a cohort study including 187,968 type 2 diabetes patients and 908,016 matched controls, where the result showed that South Asians with diabetes had lower adjusted risk of respiratory disease mortality (HR 0.60; 95% CI, 0.48–0.76) when compared to whites with diabetes. Besides, a cohort study of 204,533 participants (7,199 with diabetes) from England and Scotland reported that odds ratio for respiratory disease among those with diabetes was 1.25 (95% CI, 1.08–1.46).^16^

We examined the association by respiratory diseases subtypes, which showed that the risk of mortality associated with diabetes did vary across different respiratory diseases subtypes. Diabetes was associated with the risk of mortality from respiratory infection, and the significant association was consistently observed regardless of sex, age, baseline smoking status, and BMI levels. As an established risk factor for infections including common infection of respiratory tract, several studies have showed that diabetic patients had 1.5- to 2.5-fold increased risk of mortality due to infection compared with populations without diabetes.^17^^,^^18^ Thus, the prevention and control of respiratory infection should be addressed more intensively in the management of diabetic patients.^19^ However, our study failed to find significant association between diabetes and the risk of mortality from COPD and other respiratory diseases, which was different from the findings in the cohort study of Chinese populations.^9^ The number of deaths from COPD (1,941) in the Chinese cohort was much larger than that in the current study, which could be an explanation for the inconsistent results between the two studies. Since COPD has a long disease process, with diabetes often coexisting, as well as the complicated underlying mechanism about the relationship between the two diseases,^20^ the causal relationship between diabetes and COPD mortality remains to be further explored.

In the current analysis, we found that baseline history of diabetes was significantly associated with risk of mortality from total respiratory diseases among women but not for men, where sex may significantly modify the association. Gordon-Dseagu et al^16^ also reported that an increased odds of mortality from respiratory diseases was only observed among women rather than men after adjusting for age. The underlying mechanism about the sex-specific association between diabetes and the risk of mortality from total respiratory diseases needs further study.

Moreover, we conducted a subgroup analysis to explore the association between diabetes and respiratory disease mortality according to different age groups, and found that the associations between diabetes and the risk of mortality from total respiratory diseases and respiratory infection were stronger among participants aged 40–64 years than those aged 65–79 years. A previous study also showed a significant interaction between age and type 2 diabetes for respiratory disorders, where the association between diabetes and respiratory disorders was stronger for younger age classes.^21^

In the subgroup analysis based on baseline smoking status, we observed a significant association between diabetes and the risk of mortality due to total respiratory diseases among never smokers rather than ever smokers, and the interaction between smoking and diabetes was significant. The relationship between diabetes, smoking, and the risk of respiratory diseases was complicated. Previous studies have indicated that both smoking and diabetes were risk factors for death from respiratory diseases.^22^^,^^23^ In addition, there was significant interaction between smoking and diabetes for the risk of respiratory diseases.^24^

We also conducted stratified analysis according to baseline BMI levels and found that the interaction between diabetes and BMI was not statistically significant, although diabetes was associated with the risk of mortality among participants with BMI ≥25 kg/m^2^ but not among those with BMI <25 kg/m^2^. Further, the significant association between diabetes and respiratory infection was consistently observed regardless of the BMI category. A previous study also showed the association between diabetes and the risk of mortality from respiratory diseases was not modified by overweight/obesity.^16^

There are several potential mechanisms for the association between diabetes and the risk of respiratory disease mortality, such as infection and inflammation, hyperglycemia, oxidative stress, and decreased lung function.^25^^–^^29^ Several studies showed that common indicators of inflammation could predict the development of both diabetes and COPD.^25^^–^^27^ In addition, hyperglycemia may harm immune function, antioxidant systems, and complement activation, thus increasing the risk of deaths due to infection.^28^ Moreover, as a target organ for diabetes, the function of the lungs may be impaired by diabetic microangiopathy.^29^ The potential relationship between diabetes and respiratory disease mortality still needs more research in the future.

In order to evaluate the potential effect of competing events of nonrespiratory-specific mortality on the association between diabetes and mortality from respiratory diseases, we adopted Fine and Gray’s sub-distribution hazard model^30^ to calculate subdistribution hazards (eTable 1, eTable 2, eTable 3, and eTable 4). We found that there was significant association between diabetes and the risk of mortality due to respiratory infection in women (HR 1.54; 95% CI, 1.14–2.08) although the effect was smaller than that estimated using Cox regression models. Moreover, for participants aged 40–64 years, diabetic patients had an increased risk of mortality from total respiratory diseases (HR 1.59; 95% CI, 1.17–2.17) and respiratory infection (HR 2.24; 95% CI, 1.55–3.23). In addition, we observed significant association between diabetes and mortality from respiratory infection among never smokers (HR 1.45; 95% CI, 1.07–1.96). Further, a negative association between diabetes and COPD was detected among participants with BMI <25 kg/m^2^ (HR 0.47; 95% CI, 0.26–0.85), which was similar with the result calculated using the Cox proportional hazards model (HR 0.56; 95% CI, 0.31–1.03). Thus, the results calculated using Fine and Gray’s sub-distribution hazard model were broadly consistent with those in the current analysis.

The present study included a large sample size of the Japanese population to evaluate the association between diabetes and the risk of mortality from different types of respiratory diseases, enabling us to conduct several stratified analyses by sex, age, smoking status, and BMI levels. There are still several limitations for our study. First, the definition of diabetes was based on the self-reported questionnaire, and diabetes status during follow-up was not available, which may cause misclassification of diabetes. In addition, the control status of diabetes may influence the association between diabetes and mortality from respiratory diseases, but the information was not available in the current study. Therefore, future studies are needed to verify our findings considering the control of diabetes. Another limitation of the study was that the information of other potential residual confounding factors, such as vaccination history of pneumonia and flu, as well as socioeconomic status, including income, was unavailable. Also, the number of deaths from COPD was relatively small in our study, which may result in limited statistical power.

In conclusion, the prospective cohort study revealed that diabetes was significantly associated with increased mortality from total respiratory diseases among women, but the association was of borderline statistical significance in men. However, diabetes was associated with mortality due to respiratory infection among both men and women. The study shows prevention and control of respiratory diseases, especially respiratory infection, should be paid more attention among people with diabetes in clinical and public health practice.

## APPENDIX A. SUPPLEMENTARY DATA

eTable 1. Sex-specific and age-adjusted and multivariable hazard ratios of mortality from respiratory diseases according to baseline history of diabetes calculated using Fine and Gray’s sub-distribution hazard model

eTable 2. Multivariable hazard ratios of mortality from respiratory diseases according to baseline history of diabetes, stratified by age groups calculated using Fine and Gray’s sub-distribution hazard model

eTable 3. Age-adjusted and multivariable hazard ratios of mortality from respiratory diseases according to baseline history of diabetes, stratified by smoking status calculated using Fine and Gray’s sub-distribution hazard model

eTable 4. Age-adjusted and multivariable hazard ratios of mortality from respiratory diseases according to baseline history of diabetes, stratified by body mass index calculated using Fine and Gray’s sub-distribution hazard model
