# Supplementary material for: Diabetes and Mortality From Respiratory Diseases: The Japan Collaborative Cohort Study
Source: J Epidemiol. 2020 Oct 5;30(10):457–63. doi: 10.2188/jea.JE20190091 (PMC7492709; doi:10.2188/jea.JE20190091)
Supplement: Supplementary file 1 [file je-30-457-s001.pdf]

eTable 1. Sex-specific and age-adjusted and multivariable hazard ratios of mortality from respiratory diseases according to baseline history of diabetes calculated using Fine and Gray's sub-distribution hazard model

	Men			Women		
	Baseline history of diabetes			Baseline history of diabetes		
	Yes	No	*P* trend	Yes	No	*P* trend
Total respiratory diseases						
HR (95% CI)[a]	0.94 (0.77–1.14)	1.00	0.50	1.16 (0.89–1.51)	1.00	0.29
HR (95% CI)[b]	0.95 (0.78–1.16)	1.00	0.63	1.23 (0.94–1.61)	1.00	0.13
Respiratory infection						
HR (95% CI)[a]	1.09 (0.86–1.38)	1.00	0.47	1.41 (1.05–1.89)	1.00	0.02
HR (95% CI)[b]	1.12 (0.88–1.42)	1.00	0.37	1.54 (1.14–2.08)	1.00	0.01
COPD						
HR (95% CI)[a]	0.69 (0.42–1.14)	1.00	0.15	–	–	-
HR (95% CI)[b]	0.69 (0.42–1.15)	1.00	0.16	–	–	-
Other respiratory diseases						
HR (95% CI)[a]	0.73 (0.47–1.15)	1.00	0.18	0.84 (0.45–1.59)	1.00	0.60
HR (95% CI)[b]	0.77 (0.49–1.22)	1.00	0.27	0.86 (0.46–1.60)	1.00	0.63

CI, confidence interval; COPD, chronic obstructive pulmonary disease; HR, hazard ratio.

[a] Adjusted for age

[b] Adjusted for age, educational level, body mass index, smoking, alcohol consumption, sports activity, walking time, and family history of diabetes

eTable 2. Multivariable hazard ratios of mortality from respiratory diseases according to baseline history of diabetes, stratified by age groups calculated using Fine and Gray's sub-distribution hazard model

	Age <65 years			Age ≥65 years		
	Baseline history of diabetes			Baseline history of diabetes		
	Yes	No	*P* trend	Yes	No	*P* trend
Total respiratory diseases						
HR (95% CI)[a]	1.59 (1.17–2.17)	1.00	<0.01	1.02 (0.85–	1.00	0.84
Respiratory infection						
HR (95% CI)[a]	2.24 (1.55–3.23)	1.00	<0.001	1.20 (0.96–	1.00	0.11
COPD						
HR (95% CI)[a]	0.73 (0.27–2.00)	1.00	0.54	0.64 (0.37–	1.00	0.12
Other respiratory diseases						
HR (95% CI)[a]	0.96 (0.47–1.95)	1.00	0.90	0.81 (0.53–	1.00	0.35

CI, confidence interval; COPD, chronic obstructive pulmonary disease; HR, hazard ratio.

[a] Adjusted for sex, educational level, body mass index, smoking, alcohol consumption, sports activity, walking time, and family history of diabetes

eTable 3. Age-adjusted and multivariable hazard ratios of mortality from respiratory diseases according to baseline history of diabetes, stratified by smoking status calculated using Fine and Gray's sub-distribution hazard model

	Never smokers			Ever smokers [a]		
	Baseline history of diabetes			Baseline history of diabetes		
	Yes	No	*P* trend	Yes	No	*P* trend
Total respiratory diseases						
HR (95% CI)[b]	1.26 (0.98–1.63)	1.00	0.08	0.89 (0.72–1.10)	1.00	0.29
HR (95% CI)[c]	1.29 (0.99–1.68)	1.00	0.06	0.92 (0.74–1.15)	1.00	0.47
Respiratory infection						
HR (95% CI)[b]	1.39 (1.04–1.86)	1.00	0.03	1.09 (0.85–1.41)	1.00	0.50
HR (95% CI)[c]	1.45 (1.07–1.96)	1.00	0.02	1.12 (0.86–1.46)	1.00	0.39
COPD						
HR (95% CI)[b]	0.86 (0.31–2.33)	1.00	0.76	0.56 (0.31–0.99)	1.00	0.05
HR (95% CI)[c]	0.82 (0.29–2.33)	1.00	0.72	0.59 (0.33–1.06)	1.00	0.08
Other respiratory diseases						
HR (95% CI)[b]	0.98 (0.53–1.80)	1.00	0.95	0.69 (0.42–1.14)	1.00	0.15
HR (95% CI)[c]	0.96 (0.52–1.77)	1.00	0.90	0.73 (0.44–1.22)	1.00	0.23

CI, confidence interval; COPD, chronic obstructive pulmonary disease; HR, hazard ratio.

[a] Former smokers and current smokers

[b] Adjusted for age

[c] Adjusted for age, sex, educational level, body mass index, alcohol consumption, sports activity, walking time, and family history of diabetes

eTable 4. Age-adjusted and multivariable hazard ratios of mortality from respiratory diseases according to baseline history of diabetes, stratified by body mass index calculated using Fine and Gray's sub-distribution hazard model

	Body mass index <25 kg/m^2			Body mass index ≥25 kg/m^2		
	Baseline history of diabetes			Baseline history of diabetes		
	Yes	No	*P* trend	Yes	No	*P* trend
Total respiratory diseases						
HR (95% CI)[a]	1.03 (0.85–1.23)	1.00	0.79	1.24 (0.83–1.86)	1.00	0.30
HR (95% CI)[b]	0.95 (0.78–1.14)	1.00	0.56	1.26 (0.84–1.91)	1.00	0.26
Respiratory infection						
HR (95% CI)[a]	1.22 (0.99–1.52)	1.00	0.07	1.37 (0.85–2.23)	1.00	0.20
HR (95% CI)[b]	1.16 (0.93–1.45)	1.00	0.19	1.40 (0.86–2.29)	1.00	0.17
COPD						
HR (95% CI)[a]	0.55 (0.30–1.01)	0.29	0.05	1.76 (0.62–5.00)	1.00	0.29
HR (95% CI)[b]	0.47 (0.26–0.85)	1.00	0.01	1.79 (0.59–5.50)	1.00	0.31
Other respiratory diseases						
HR (95% CI)[a]	0.82 (0.53–1.25)	1.00	0.36	0.70 (0.26–1.93)	1.00	0.50
HR (95% CI)[b]	0.77 (0.50–1.19)	1.00	0.24	0.71 (0.25–1.99)	1.00	0.51

CI, confidence interval; COPD, chronic obstructive pulmonary disease; HR, hazard ratio.

[a] Adjusted for age

[b] Adjusted for age, sex, educational level, smoking, alcohol consumption, sports activity, walking time, and family history of diabetes
